# Supplementary figures and images for: The centrosomin CM2 domain is a multi-functional binding domain with distinct cell cycle roles
Source: PLoS One. 2018 Jan 9;13(1):e0190530. doi: 10.1371/journal.pone.0190530 (PMC5760045; doi:10.1371/journal.pone.0190530)

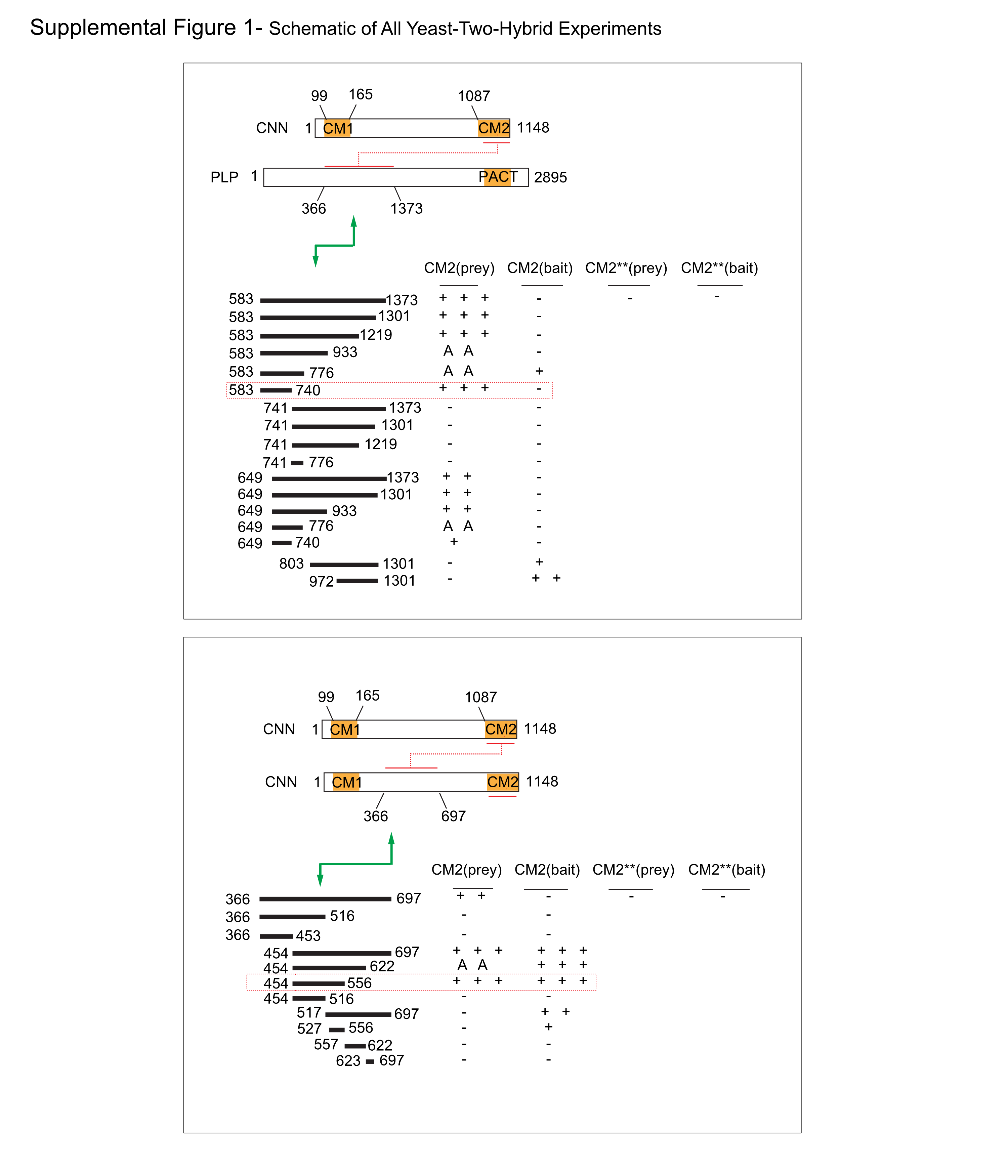

Supplement: S1 Fig — (TIFF) [file pone.0190530.s001.tiff]

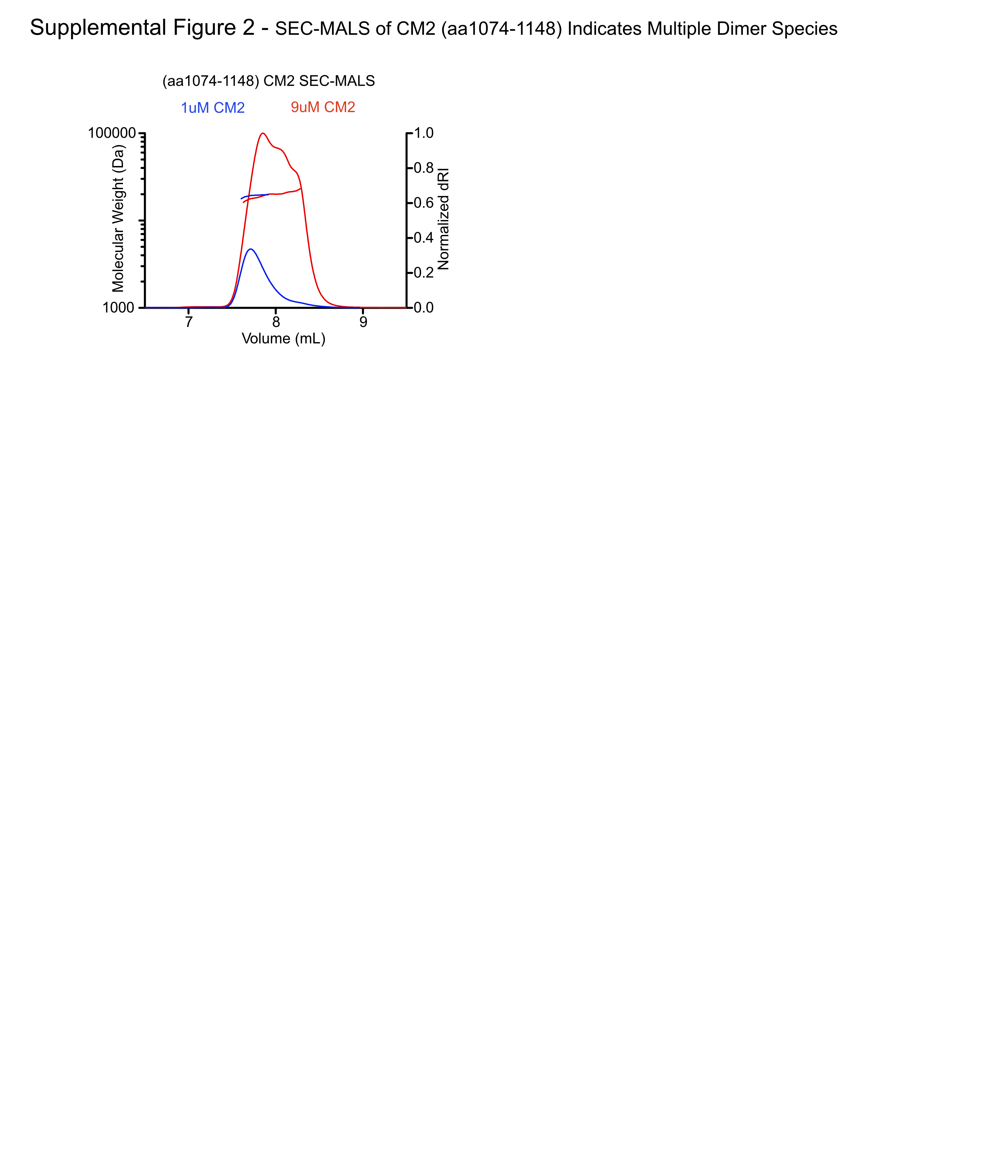

Supplement: S2 Fig — CM2 construct used for NMR appears dimeric by SEC-MALS analysis. At 1μM (blue) this construct appears a predominately, as a single dimeric peak with a calculated molecular weight of 19.2kDa while the predicted monomer weight is 9kDa. At 9μM (red) this same construct appears as three or more not fully resolved peaks, however, the molecular weight across all these peaks appears to remain dimeric. (TIFF) [file pone.0190530.s002.tiff]

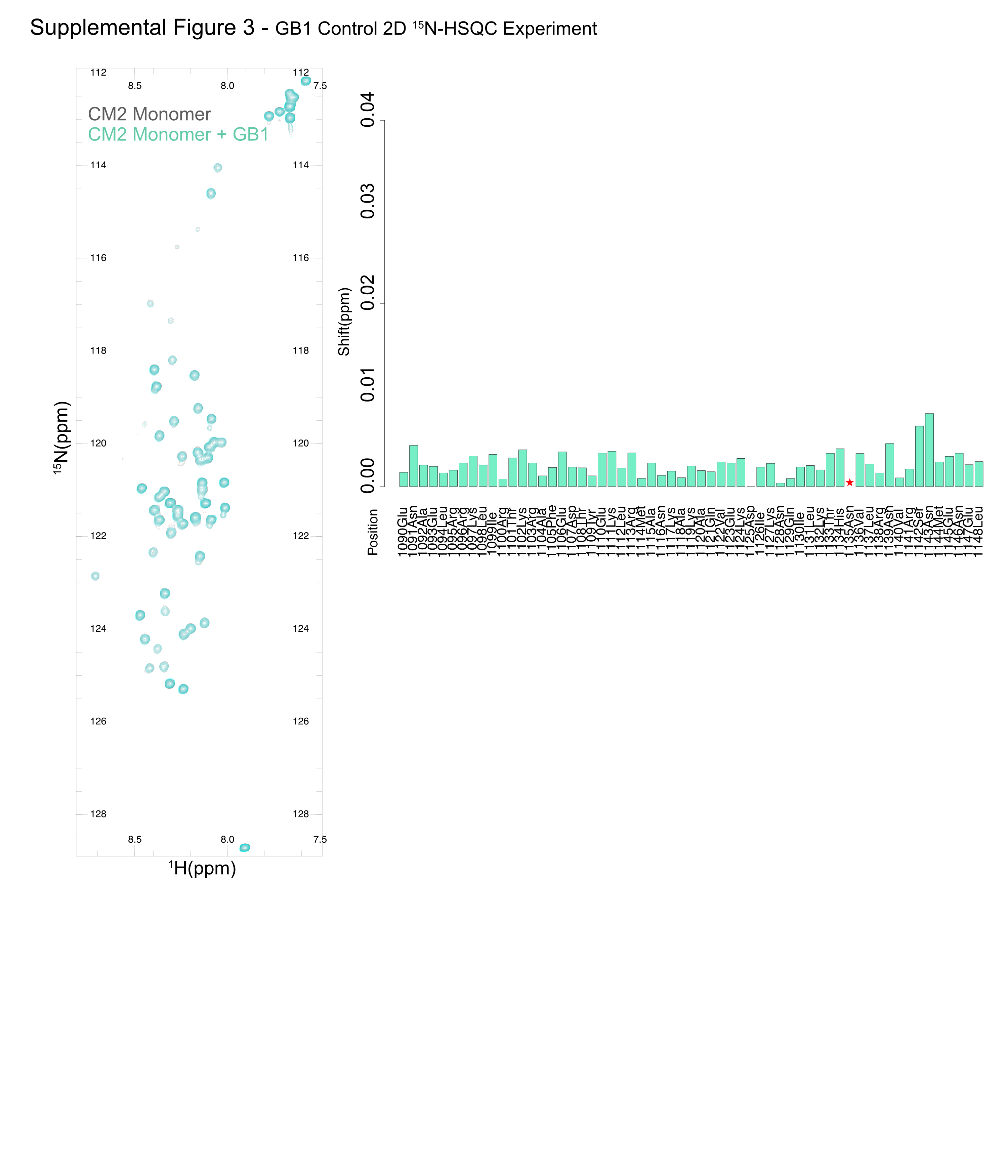

Supplement: S3 Fig — (A) HSQC spectra from a control experiment of monomeric CM2 (gray) and monomeric CM2 with 150μM GB1 (cyan) overlaid. All the peaks appear unperturbed in the presence of GB1. B) Quantitation of peak shifts on addition of GB1 with the same y-axis scaling as for the GB1-CNNLZ and GB1-PLPMD data in Fig 2D and 2E. (TIFF) [file pone.0190530.s003.tiff]

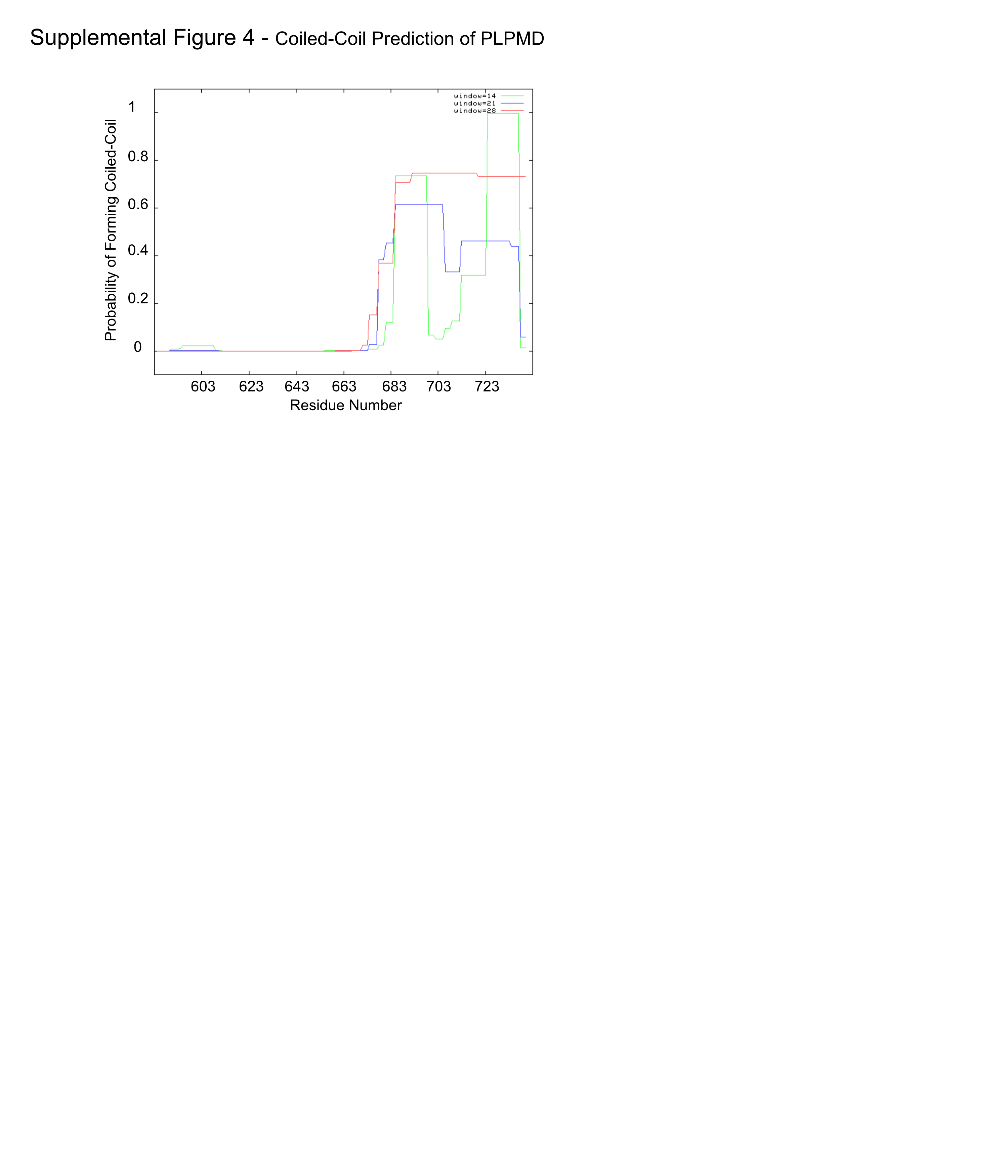

Supplement: S4 Fig — Coiled-coil prediction of PLPMD from the COILS server shows high probability of a coiled coil secondary structure in the carboxyl region of PLPMD. (TIFF) [file pone.0190530.s004.tiff]

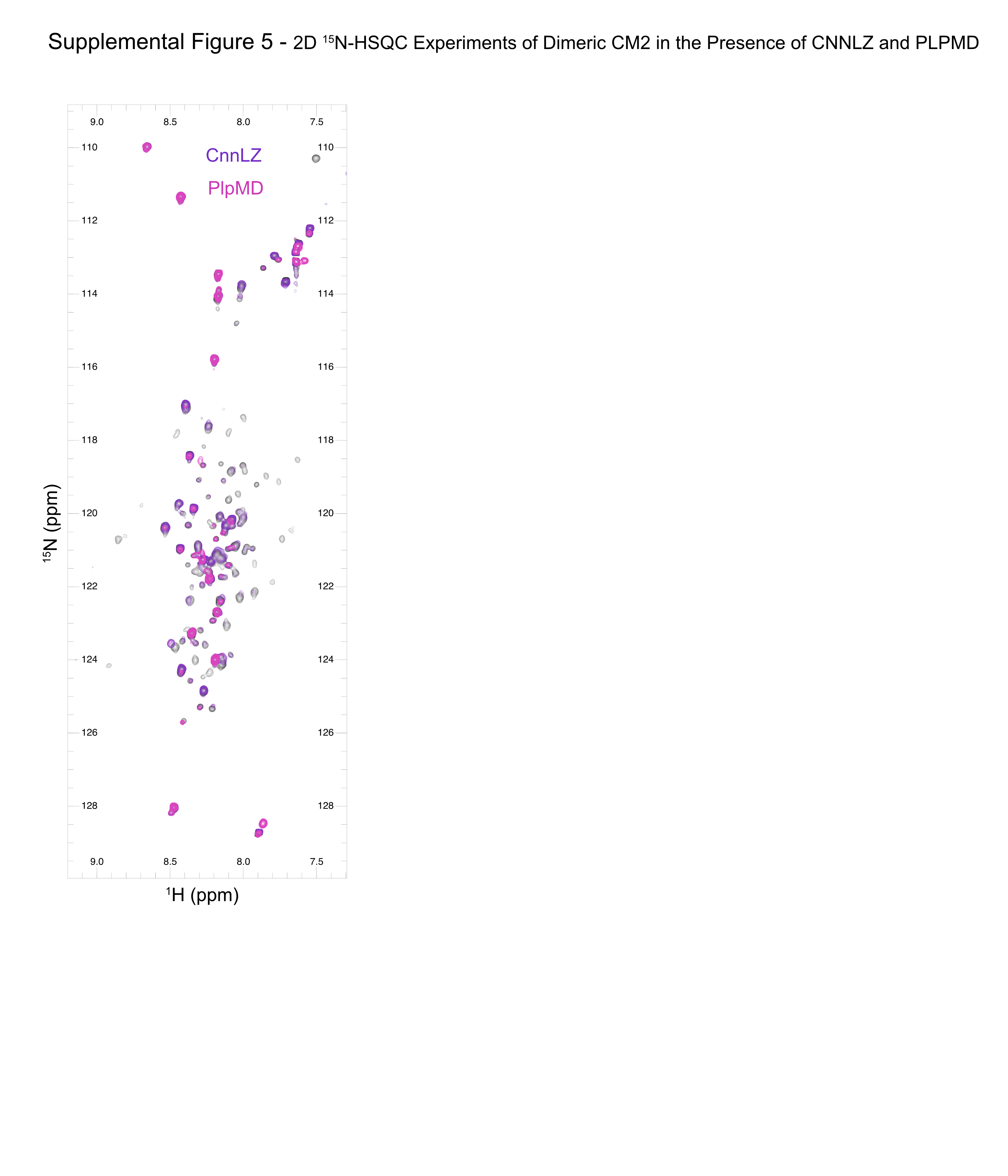

Supplement: S5 Fig — HSQC spectra (gray) of dimeric CM2 with low intensity peaks at lower (<8ppm) proton resonances indicative of multiple species. Overlaid are dimeric CM2 with addition of 75μM CNNLZ (purple) and 100μM PLPMD (pink) that exhibit reduced intensities on a significant number of peaks. All spectra are contoured and scaled identically. (TIFF) [file pone.0190530.s005.tiff]

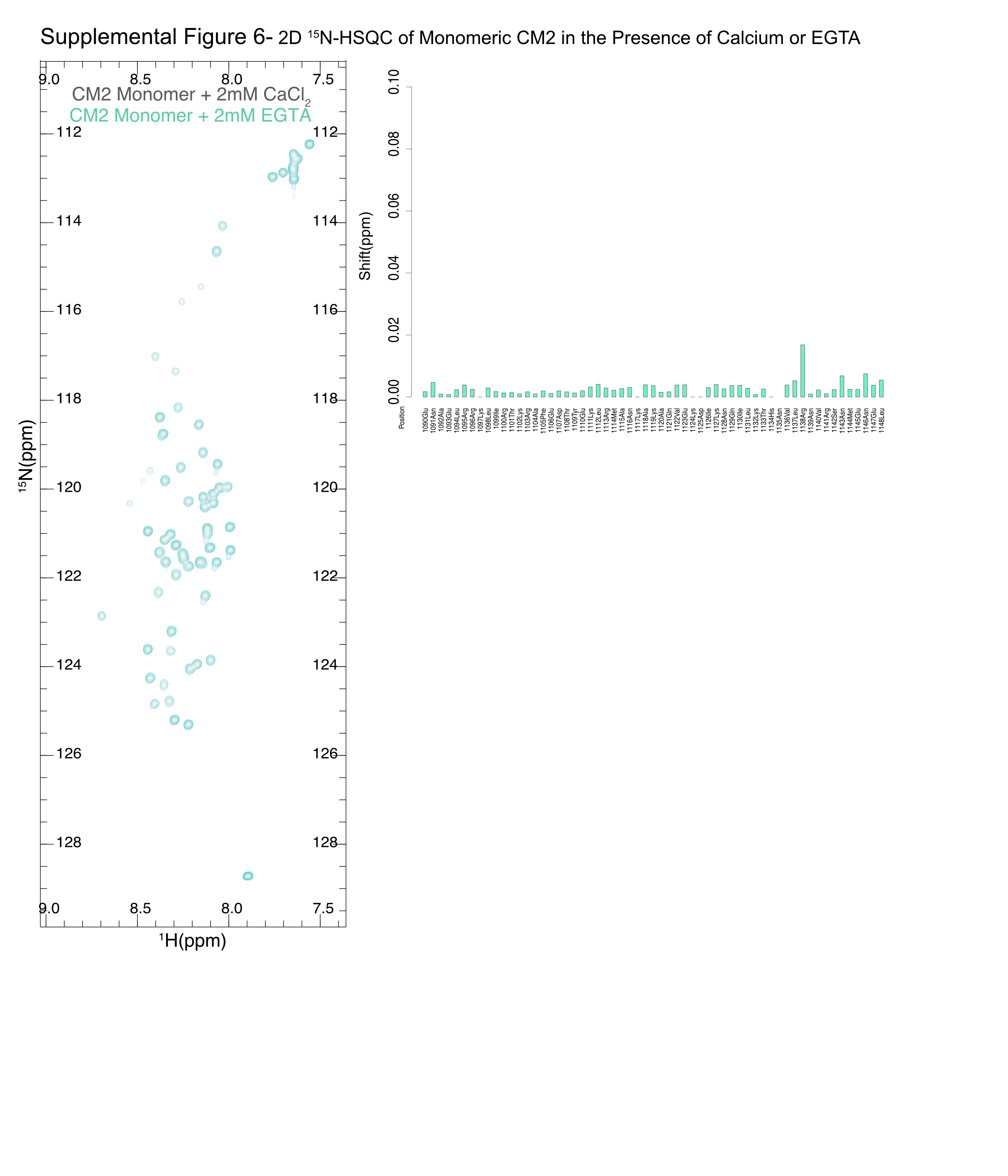

Supplement: S6 Fig — Overlay of the HSQC spectra of monomeric CM2 in the presence of 2mM CaCl2 (gray) or in the presence of 2mM EGTA (cyan). Visual inspection of the spectra as well as quantification of the combined chemical shifts between the two shows that there are no appreciable difference to the spectra. (TIFF) [file pone.0190530.s006.tiff]

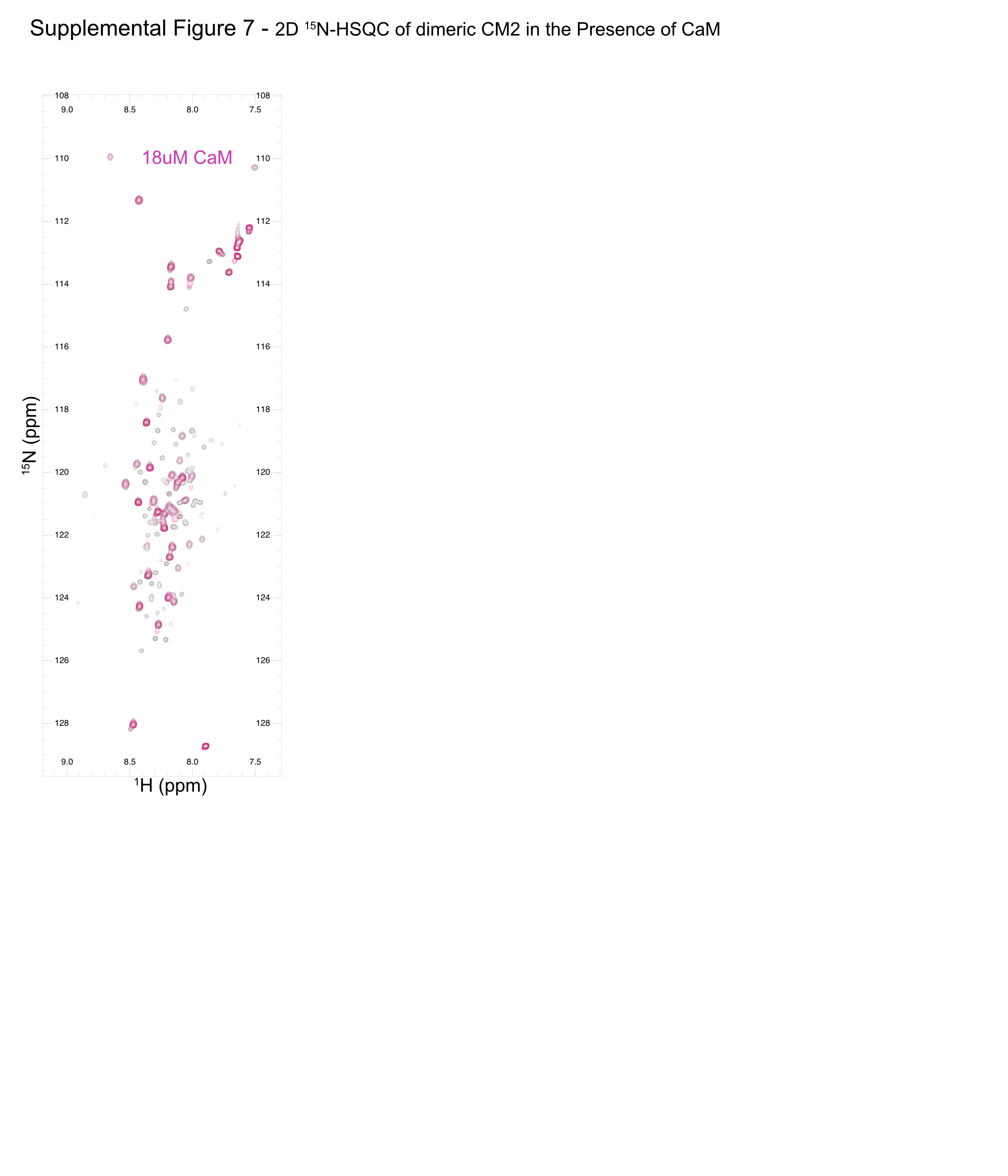

Supplement: S7 Fig — Overlay of the HSQC spectra of dimeric CM2 (gray) and dimeric CM2 with the addition of 18μM CaM (pink) in the presence of 2mM CaCl2. Both spectra are contoured and scaled identically. The intensities of several peaks are reduced dramatically with the addition of CaM and new peaks also appear in the presence of CaM. (TIFF) [file pone.0190530.s007.tiff]
